# Supplementary material for: Angelica sinensis polysaccharide as potential protectants against recurrent spontaneous abortion: focus on autophagy regulation
Source: Front Med (Lausanne). 2025 Jan 15;12:1522503. doi: 10.3389/fmed.2025.1522503 (PMC11774876; doi:10.3389/fmed.2025.1522503)
Supplement: Supplementary material S1 — The main instruments used during the LC-MS process, along with their models/specifications and manufacturers. [file Supplementary_file_1.zip › Metabolomics sequencing data FC1.2/8.代谢通路分析/Exposure_Ctrl/up/气泡图-p小于0.05.pdf]

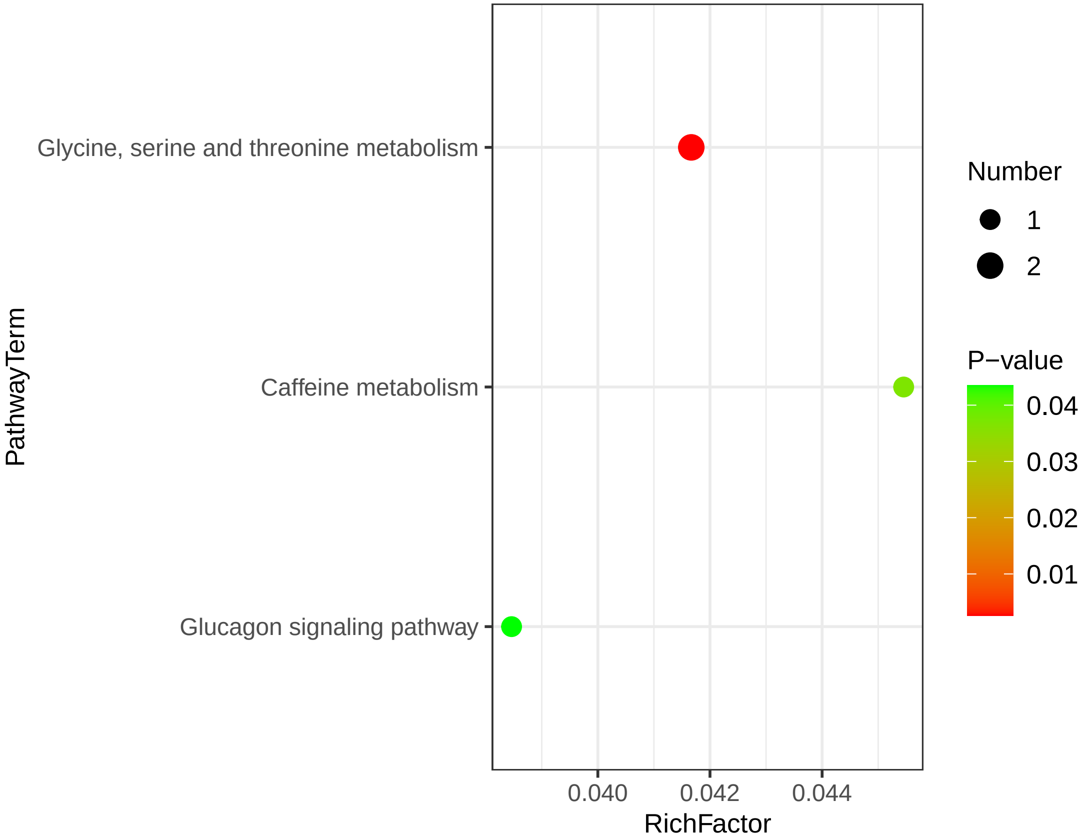

Glycine, serine and threonine metabolism
Caffeine metabolism
Glucagon signaling pathway
PathwayTerm
0.040
0.042
0.044
RichFactor
Number
1
2
P−value
0.04
0.03
0.02
0.01
